# Masseter muscle flap for reconstruction of intra-oral defects in patients with early cancer of posterior-inferior parts of the oral cavity

**DOI:** 10.1016/j.bjorl.2020.10.010

**Published:** 2020-11-16

**Authors:** B.C. Rajani, Hoda Nadimul, Ghosh Subhabrata, K.S. Sabitha, Annavarjula Vinitha, B. Vasantha Dhara

**Affiliations:** Kidwai Memorial Institute of Oncology, Department of Oral Oncology, Bengaluru, Karnataka, India

**Keywords:** Oral cancer, Masseter muscle flap, Trismus, Deviation on mouth opening

## Abstract

**Introduction:**

Early carcinomas of the oral cavity in the posterior-inferior regions poses a challenge for reconstruction due to the lack of muscle support underneath and the limited space available to use some of the frequently-used flaps.

**Objective:**

This study was done to evaluate the efficacy of the superiorly based masseter muscle flap in reconstruction of intra-oral post- ablation defects in patients with early oral carcinoma of the posterior-inferior part of the oral cavity.

**Methods:**

A superiorly based masseter muscle flap were used to reconstruct the post-surgical intra- oral defect in 60 patients with early squamous cell carcinoma (T < 4 cm) of the posterior-inferior part of the oral cavity. The patients were followed up at 1-week and 1-month postoperatively to check for flap viability, complications, change in mouth opening and deviation of the mandible on mouth opening. To rule out any recurrence in the oral cavity masseter flaps, the patients were followed up for 1 year.

**Results:**

The flap was viable in all patients and underwent mucosalization. 7/60 patients had postoperative infections, while 2/60 patients developed an oro-cutaneous fistula which required a secondary corrective procedure. The mean ± standard deviation of change in mouth opening at 1 week postoperatively was +1.917 ± 3.36 mm, which increased to +2.633 ± 2.95 mm at 1 month after surgery. The Friedman test revealed that there was a statistically significant change in mouth opening from preoperative period to the1 week and 1 month postoperative periods (p = 0.000). Female patients showed better improvement in mouth opening postoperatively. The ipsilateral deviation of the mandible on mouth opening was between 0–5 mm in 39 patients, 5–10 mm in 17 patients and more than 10 mm in 4 patients. There were no recurrences noted in the masseter flaps used.

**Conclusion:**

The study infers that the superiorly based masseter muscle flap is a reliable method for reconstruction in early oral cancer patients yielding good functional results and acceptable cosmesis with nominal postoperative complications.

## Introduction

Resection of oral malignancies with safe margins lead to deficits in various functions like speech, swallowing, mouth opening etc.^1^, ^2^, ^3^ Thus adequate reconstruction of the post- resection defect becomes a necessity and a challenge in order to restore both function and esthetics.^4^ Several local, regional and free flaps are being used presently. The choice of the reconstruction method is chiefly dependent on various factors like the size of the post- resection defect, age of the patient, presence of any comorbidities, whether previous surgeries like ipsilateral neck dissections have been done or not, surgeon’s preference etc.^2^, ^5^

Despite the current advancements in microvascular surgeries, the use of local and regional flaps remains a preferred option for a surgeon considering the simplicity of the surgical technique and favorable results. The masseter muscle is a muscle of mastication which helps in elevation of the mandible and is one of the strongest muscles of the human body. Its use in reconstruction of oral defects was first advocated by Tiwary and Snow and Langdon in 1989.^2^, ^3^ Its use for reconstruction of oropharyngeal defects was first documented by Conley and Gullane in 1978.^6^ This muscle has also been widely used in cases of facial palsies for reanimation purposes.^7^

There are very few studies in the literature dealing with this unique mode of reconstruction of oral cavity following carcinoma resection in posterior-inferior areas. Thus, the purpose of this study is to evaluate the efficacy of superiorly based masseter muscle flaps in reconstruction and repair of intraoral surgical defects in patients with early cancer of theposterior-inferior parts of the oral cavity.

## Methods

A prospective study was conducted where 60 patients who reported to our institute between January, 2019 and June, 2019 with histopathologically proven carcinoma of the posterior-inferior part of the oral cavity were included, and who subsequently underwent reconstruction using an ipsilateral superiorly based masseter muscle flap. Approval by the Ethics Committee of institutional review board (ref. nº 324–2019) and informed consent was obtained from the patients who were included in the study. In order to be included in the study, the tumor of the oral cavity had to be less than 4 cm in size (early primary lesion), located in posterior half of buccal mucosa, lower gingivo-buccal sulcus or retromolar trigone, abutting or involving the mandible, necessitating marginal mandibulectomy or segmental resection or hemi-mandibulectomy as per established principles of oncological resection. It was ensured by reviewing preoperative CT Scans that the masseter muscle was not involved by the tumor. The patient should not have undergone any previous radiotherapy or chemotherapy and/or should not have any history of neuromuscular or muscle degenerating disorders.

### Surgical technique

During or after the resection of the primary tumor, the cheek flap was raised until the masseter muscle is exposed.

**Fig. 1:**
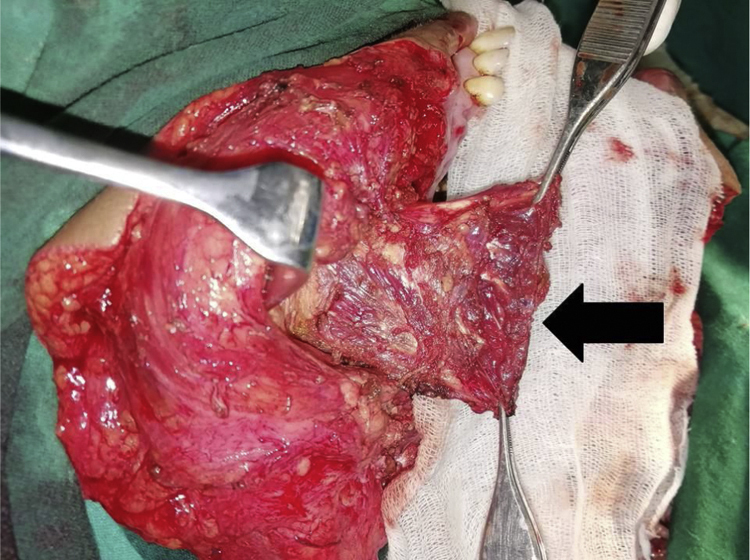
The superiorly based masseter muscle flap (arrow) is harvested for reconstruction.
- The posterior border of the muscle was used to reconstruct the retro-mandibular raphe and retromolar trigone area by suturing the masseter muscle to the remaining cut ends of the pterygoid muscles (in case of segmental or hemimandibulectomy) or the mucosa adjoining the third molars.
- The inferior cut end of the muscle (severed from its insertion into angle of mandible) was sutured with the mylohyoid muscle and/or mucosal membrane of the floor of the mouth remaining after the removal of the primary tumor to reconstruct the posterior-inferior floor of the mouth and sulcus.
- The anterior post resection defect (if present) was closed primarily using transpositional mucosal flaps from buccal mucosa, floor of the mouth or tongue.

- The fascia of the masseter over the angle of the mandible was incised and dissected free along with the cheek flap to preserve the branches of facial nerve.
- The muscle was dissected free along its posterior margin from the parotid gland until the zygomatic arch. The origin of the muscle from the posterior part of zygomatic arch was severed so that the flap could be rotated from a posterior-inferior direction to an antero-inferior direction (Fig. 1).

Postoperatively the patients were treated with aggressive passive and active mouth opening exercises.

**Fig. 2:**
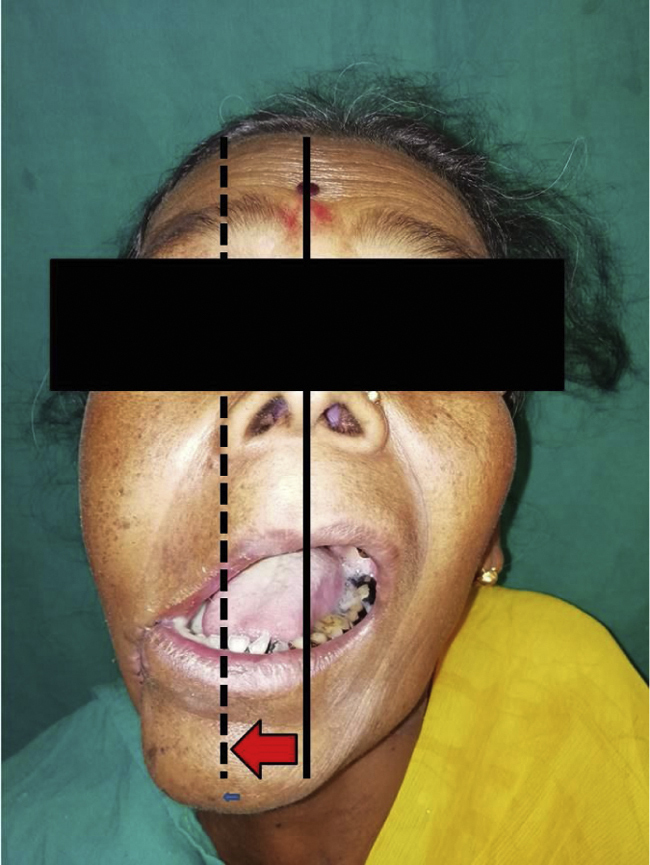
Ipsilateral deviation of mandible on mouth opening (continuous line – actual midline, dotted line – midline of mandible).

Postoperatively, the patients were followed up at 1 week and 1 month after surgery and were examined for postoperative complications like infection, fistula, or postoperative change in mouth opening (Change in mouth opening at 1 week after surgery was calculated by obtaining the difference between mouth opening [MO] at 7th postoperative day and preoperative MO and likewise the change in MO at 1 month postop was also calculated) and deviation of mandible on mouth opening (lateral deviation if any, from the imaginary line passing in between the right and left maxillary central incisors) (Fig. 2). All measurements of magnitude of MO and deviation of mandible on MO were done using Vernier calipers. All the patients were followed up for 1 year after the surgeries to check for any residual tumors or recurrences in the masseter flaps used.

### Statistical analysis

The Shapiro-Wilk test of normality was done on the preoperative, 1 week postop and 1 month postop mouth opening values of all patients, and the Friedman test was used to test the significance of change in mouth opening of the patients from preoperative time to 1 week postop and 1 month postop. Pearson correlation analysis was done to test any association between mandibular deviation on mouth opening and gender of patient and between mouth opening at the end of 1-week and 1-month and gender of the patient. Results were considered to be significant at a 5% critical level (*p* < 0.05). Statistical analysis was done using IBM SPSS Statistics (ver. 21.0; IBM, Armonk, NY, USA).

## Results

Out of 60 patients included in this study, 41 (68.3%) were females and 19 (31.7%) were males, 5/60 patients were between the age group of 30–40 years, 26/60 patients were between the age group of 40–50 years, 16/60 patients were in the age group of 50–60 years and 13/60 patients were aged more than 60 years, and 36/60 patients had comorbidities present like diabetes and hypertension (Table 1).

**Table 1:** Descriptive statistics of patients undergoing masseter flap reconstruction.

Gender	Male	18
	Female	42
Age	30–40 years	5
	40–50 years	26
	50–60 years	16
	60+ years	13
Comorbidities	Present	36
	Absent	24
Infection	Present	07
	Absent	53
Fistula	Present	02
	Absent	58
Secondary correction	Required	02
	Not required	58
Recurrence in flaps	Present	0
	Absent	60

**Fig. 3:**
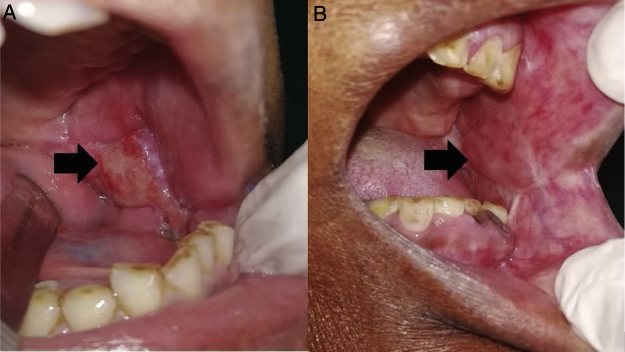
Masseter flap at (a) 1-month post op and (b) 1-year post op showing mucolisation (arrow).

In all the patients the flap was viable all throughout and underwent mucosalisation over a period of time. In 7 patients postoperative infections were present, while 2/60 patients developed an orocutaneous fistula which required a secondary procedure for correction (Table 1). At 1 week postop in 18/60 patients, the mouth opening was reduced when compared to preoperative MO, while in 32/60 patients the increase in postop MO when compared to preop MO was in between 0–5 mm. In the remaining 10 patients an increase in MO of 5–10 mm was noticed (mean ± standard deviation: 33.05 ± 3.48 mm). When readings were taken at 1 month postop, only 9/60 patients had reduced MO in comparison to their preoperative values, while 39/60 patients saw an increase in 0–5 mm in MO postoperatively while the number of patients whose increase in MO was between 5–10 mm increased to 12 (mean ± standard deviation: 33.93 ± 3.09 mm) (Table 2, Fig. 3). The mean ± standard deviation of change in mouth opening at 1 week postop was +1.917 ± 3.36 mm which increased to +2.633 ± 2.95 mm at 1 month postop (Table 2). The Shapiro-Wilk normality test showed that postoperative mouth opening values were statistically significant and were not normally distributed (MO at 1 week postop: *p* = 0.023, MO at 1-month post op: *p* = 0.019). Friedman test revealed that there was a statistically significant change in mouth opening from the preoperative period to 1 week and 1 month postoperative periods (*p* = 0.000). When the association between gender and change in mouth opening was studied, it was seen that female patients showed statistically significant positive correlation to change in mouth opening postoperatively both at 1 week postop (*p* = 0.198) and 1-month postop (*p* = 0.111) (Table 2). The ipsilateral deviation of mandible on mouth opening was in between 0–5 mm in 39 patients, 5–10 mm in 17 patients and more than 10 mm in 4 patients (mean ± standard deviation: 4.917 ± 2.76 mm). When association between gender and deviation in mouth opening was tested, the males showed a predilection of having more mandibular deviation on mouth opening than females (*p* = 0.005) (Table 3). There were no residual tumors or recurrences noted in the masseter flaps used in any of the 60 patients (Table 1).

**Table 2:** Change in mouth opening post operatively (1-week vs. 1-month).

Change in mouth opening	1st week post op (1st week post-op mo – pre-op mo)	1-month post op (1-month post-op mo – pre-op mo)
Reduction in MO	18	9
0–5 mm	32	39
5 to 10 mm	10	12
Mean ± Standard deviation of MO	+1.917 ± 3.36 mm	+2.633 ± 2.95 mm
Correlation with gender	p = 0.198 (positive correlation with females)	p = 0.111 (positive correlation with females)
Statistical significance (Friedman test)	p = 0.000	

MO, Mouth Opening.

**Table 3:** Ipsilateral deviation of mandible on mouth opening.

Magnitude of ipsilateral deviation of mandible	Number of patients
0–5 mm	39
5–10 mm	17
10 mm +	4
Mean ± standard deviation	4.917 ± 2.76 mm
Correlation with gender	p = 0.005 (positive correlation with male)

## Discussion

Post- ablation defects of the posterior-inferior part of oral cavity which are small to moderate in size resulting from a surgery of an early tumor in oral cavity present a challenge to the surgeon while planning reconstruction.^1^, ^8^ The main objective of reconstruction after ablative surgery in cancer patients is to restore optimum function and esthetics following all the three fundamentals of reconstruction as stated by Wilson in 1981.^1^, ^3^, ^4^, ^9^ The post- ablative defect after excision of a tumor in the posterior-inferior part of the oral cavity, which is less than 4 cm but necessitates removal of a part or total of the mandible (as per principles of oncological resection) has a few peculiar features; first and foremost there is lack of mucosa, secondly there is deficient muscle tissue and most importantly there is limited space. As there is lack of muscle support, a primary closure or the use of skin grafts gives little assurance of a tight seal as it is prone to break down in the postoperative period.^3^ The free flaps are slightly more vulnerable to failure in reconstruction of such defects because of lack of space which results in folding of the flap and in turn venous stasis. Also, there are donor site morbidities associated with microvascular free flap reconstruction. Another important aspect is that in cases where a previous ipsilateral neck dissection has been performed, it is difficult to fin arteries and veins with which to anastomose the free flap, in which case a contralateral neck has to be operated to dissect out the feeder vessels for the free flap, thereby increasing the morbidity of the patient.^2^, ^3^, ^10^, ^11^, ^12^ Pedicled musculocutaneous flaps are too bulky for the limited space which might lead to pressure edema and necrosis.^3^, ^13^, ^14^ In case of masseter flaps there is a muscle layer that is not bulky, but which provides necessary support underneath, so that primary mucosal closure can be achieved.^4^

**Fig. 4:**
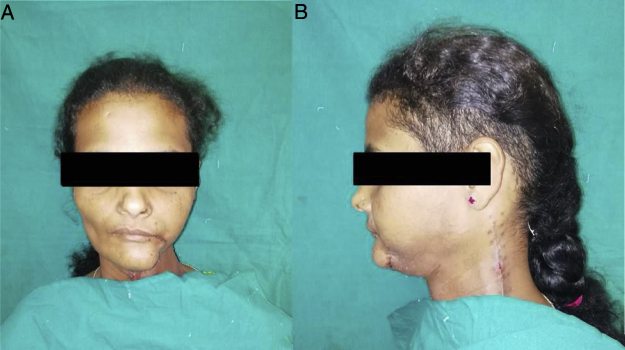
(a) Anterior profile; (b) Lateral profile of an operated case on 1-month post operatively showing nominal change in facial contour.

In our study, we included patients with small lesions which will lead to small to moderate defects post- ablation in the posterior-inferior part of the oral cavity. It was ensured that the pre-operative CT scans showed no involvement of the masseter by the tumor itself and the tissue planes were intact, making this technique of reconstruction oncologically safe and thus none of our cases showed any residual tumor or recurrence during their followup period of 1 year. Similar to studies done by Langdon and Antoniades et al.,^2^, ^5^ in our cases also the flaps were viable and they underwent epithelisation within 3–4 weeks (Fig. 3); 2 of our patients developed orocutaneous fistulas due to wound infection postoperatively. Both patients were above the age of 60 years and were known diabetics, which can be the very reason of their susceptibility to infection and hindered wound healing capacity.^15^ Though one of the chief concerns regarding this flap has been the postoperative trismus,^2^ 70% (42/60) of our patients showed an improvement in the mouth opening 1 week postoperatively and the mouth opening even improved further when reviewed 1 month after surgery. In only 30% of the patients the mouth opening was seen to reduce at 1 week postop, which can be attributed to postoperative pain and edema since after 1-month, 9 out of the 18 patients who had reduced mouth opening showed improvements.^16^ The improvement in mouth opening at 1 week and 1 month postoperatively were statistically significant. One of the reasons responsible for the improvement in postoperative mouth opening of our patients (mean mouth opening) can be attributed to the aggressive active and passive mouth opening exercises which we subjected all our patients to from the 3rd or 4th post-operative days.^17^ It was interesting to note that females showed better improvement in mouth opening postoperatively than males. This might be because the male patients had more postoperative muscle fibrosis and muscle contracture in the muscle flaps used for reconstruction which acted as a hindrance to mouth opening.^18^ However, further studies are required for a clear understanding of this aspect. Functional activities like speech and swallowing were normal and there was nominal change in the facial contour of the patients (Fig. 4). Another factor evaluated in our study was the deviation of the mandible towards the side of the surgery postoperatively (Fig. 2). This might be because of the use of buccal and mucosal transpositional flaps to attain primary closure of the anterior resection defect or postoperative scar contracture or due to hemi-mandibulectomy.^19^ In our cases a mandibular deviation of 4.917 ± 2.76 mm was noted, which is nominal and only 4 patients showed a mandibular deviation of more than 10 mm on mouth opening. Male patients showed a slightly higher tendency of having ipsilateral deviation of the mandible than female. The exact reason for this is not clear and needs further study for a better understanding; however, the small sample size of male patients in our studies may be one of the reasons.

The masseter muscle flap mainly can be of 3 types, the superiorly based (the origin from zygomatic arch is preserved), the inferiorly based (the insertion into angle of mandible is preserved) and the island flap (both origin and insertion severed, flap is pivoted around its pedicle). The masseter flap was first used as a superiorly- based cross over flap with the mandible intact but had the limitation of restriction in mobility. Nonetheless, even after marginal mandibulectomy, segmental or hemi-mandibulectomy, with the superior zygomatic insertions maintained, there is obvious limitation in flap mobility and thus is ideal to be used for reconstruction of defects of the posterior inferior area of the oral cavity.^2^, ^3^, ^20^ The inferiorly- based masseter flap is seldom used but can be effective in reconstruction of small to moderate defects of oral cavity. On the other hand, the island flap is much more pliable and has more flexibility, and thus can be also used for reconstruction of oro-pharyngeal defects.^5^

The masseter muscle flap proved to be a viable option for reconstruction in early cases of cancer in the posterior-inferior parts of the oral cavity. The procedure is not technique- sensitive and the operative time required is much less, thus reducing the total operating period and cost. It’s a onestep procedure and does not require any further correctional surgeries and also leaves no donor site defects. This mode of reconstruction is specifically useful in case of older patients with comorbidities where prolonged surgery can hamper the patient’s general health and in turn prognosis, as well as in patients who are develop a second primary in the same side of the oral cavity as their first primary. Still, we have to keep in mind the limitations of this flap like proximity to the primary tumor, inability to cover large defects and lack of mobility before choosing this as a reconstruction measure (Table 4).

**Table 4:** Advantages and disadvantages of superiorly based masseter muscle flap.

Advantages	Disadvantages
Short surgical procedure, less operating time	Limited size can be used for small to moderate defects
Not technique sensitive	Limited mobility to be used for reconstruction of posterior-inferior parts of oral cavity and oro-pharyngeal defects
Can be used in cases of second primary where previous neck dissections have been done	
No donor site morbidities	Proximity to the primary tumor
Single staged procedure	Post-operative trismus
Can be used in old patients and medically compromised patients who can’t withstand long surgical procedures	No epithelial/epidermal cover muscle susceptible to undergo fibrosis.
Acceptable cosmesis	
Good functions-speech, swallowing	
Less failure rates	

## Conclusion

We demonstrate in this study that the superiorly based masseter muscle flap is a reliable method for reconstruction in early carcinoma cases in the posterior-inferior parts of the oral cavity. Like every flap it has its set of advantages and drawbacks, but if proper patient selection is done it yields good functional results and acceptable cosmesis with nominal postoperative complications.

## Conflict of interest

The authors declare no conflicts of interest.
